# Magnitude and factors associated with medication discrepancies identified through medication reconciliation at care transitions of a tertiary hospital in eastern Ethiopia

**DOI:** 10.1186/s13104-018-3668-z

**Published:** 2018-08-03

**Authors:** Addisu Tamiru, Dumessa Edessa, Mekonnen Sisay, Getnet Mengistu

**Affiliations:** 1 Department of Pharmacology and Toxicology, School of Pharmacy, College of Health and Medical Sciences, Haramaya University, P.O. Box, 235, Harar, Oromia Ethiopia; 2 Department of Pharmacy Practice, School of Pharmacy, College of Health and Medical Sciences, Haramaya University, P.O. Box, 235, Harar, Oromia Ethiopia; 3 Department of Pharmacy, College of Medicine and Health Sciences, Wollo University, Dessie, Amhara Ethiopia

**Keywords:** Medication, Reconciliation, Discrepancy, Magnitude, Factors, Hospital care, Transition

## Abstract

**Objective:**

The aim of this study is to determine the magnitude of medication discrepancies and its associated factors at transitions in care of a Specialized University Hospital in eastern Ethiopia.

**Results:**

This study enrolled 411 patients having at least one prescription medication. For each of the patient enrolled, a medication reconciliation process was accomplished between medication use history before transition and medication orders at the transition. A total of 1027 medications were reconciled and 298 of them showed discrepancies. From such medication discrepancies, 96 (32.2%) of them were unintended discrepancies. Patients admitted to surgical ward (adjusted odds ratio {AOR} 0.27 [95% confidence interval 0.10–0.74]) and on malnutrition therapy (AOR 0.13 [0.03–0.52]) had reduced likelihoods of medication discrepancies. However, patients on cardiovascular drug therapy (AOR 5.69 [2.4–13.62]) and who were hospitalized for more than 5 days (AOR 5.69 [2.97–10.9] {5–10 days}) had significantly increased likelihoods of discrepancies. Accordingly, one-third of the medication discrepancies identified were unintentional and these discrepancies were more likely to occur with cardiovascular drugs, in medical or pediatric wards and patients hospitalized for prolonged time. Therefore, this pharmacist-led medication reconciliation indicates the potential of pharmacists in reducing drug-related adverse health outcomes that arise from medication discrepancy.

**Electronic supplementary material:**

The online version of this article (10.1186/s13104-018-3668-z) contains supplementary material, which is available to authorized users.

## Introduction

Despite multidisciplinary collaborations for patient care, high rates of medication errors associated with unintended medication discrepancies remain as challenges [1–3]. Medication errors due to such unintended discrepancies could be encountered in up to 50–70% of patients during transitions in care [4–6]. Regular medication reconciliation is a useful tool or service through which patient medication safety can be improved or maintained by reducing the potential medication errors [7–9]. To be effective, this medication reconciliation service needs some actions including cooperation, task reallocation among healthcare professionals, and implementation strategy, among others, so that adverse health consequences associated with medication use can be optimally reduced [10]. Accordingly, efficient medication reconciliation can be completed by pharmacists, physicians or nurses [11–13]. More specifically, pharmacists are among key members of the team for medication reconciliation service and this also has an opportunity for reconciliation intervention and recommendation [14, 15]. Such pharmacist-led medication reconciliation service is well tested and accepted by physicians [7, 16–19].

Medication reconciliation is a process of identifying and resolving unjustified medication discrepancy between medication use history by a patient before transition and medication orders at the transition in care for the same patient [20]. A medication discrepancy identified through such reconciliation and that cannot be justified is called unintentional discrepancy; this is considered as medication reconciliation error, occurring as omission, substitution or addition, as well as discrepant dose, dosage form, and frequency [7]. The reconciliation error rate can vary based on the cause of admission or type of medication prescribed to the patient [8]. The unintended medication discrepancy has variable degree of clinically sound risk to the patients [3]. As a consequence, medication reconciliation service needs to be critically thought as one of the key strategies to reduce risks resulting from medication errors.

Various characteristics are highlighted to predict harmful discrepancies [21]. The number of medications received by a patient [18, 22], older age, sex (woman), more frequent admission in the past 12 months and reason for patient admission were key predictors indicated in relation with potential harmful discrepancies [2, 21, 23]. In some studies, drug omission was reported as a frequent cause of unintended medication discrepancy [8, 13–15]. To this end, knowing the magnitude of medication discrepancy and its associated factors is a critical strategy in minimizing adverse health outcomes of medication discrepancies in a tertiary care hospital.

## Main text

### Method

#### Study setting, period and participants

This study was conducted in selected wards of Hiwot Fana Specialized University Hospital (HFSUH). Repeated medication reconciliation processes were accomplished during February to May, 2017. All patients admitted to medical, pediatric, surgical, and obstetrics and gynecology wards of the hospital were identified and patients who had at least one transition in care during their stay in the care were included for the processes of medication reconciliation. However, patients having transition in care but who had no at least one prescribed medication before and/or at transition were excluded.

In determination of the number of patients enrolled, a 41.4% proportion of medication discrepancy [24] and a 5% margin of error with 95% confidence level were considered. Accordingly, by adding 10% of the calculated sample size for possible methodological non-contingency, medication profile of 411 patients were identified and abstracted by the use of a customized data abstraction format prepared for this reconciliation process which included medication name, dose, dosage form, and frequency of administration. The same format was employed to register medication profile for each patient at transition so that the patient’s medication reconciliation was easily performed by comparing medication orders at the transition to the medication use history for the same patient.

#### Medication reconciliation processes and outcome variable

Details of each patient’s medication profile at transition in care were compared to the details of the patient’s medication use profile before the transition. Forms of medication discrepancy included in the analysis were omission or addition of a medication, substitution of an agent within the same therapeutic class, and change in dose, frequency, or route of administration. Accordingly, any type of discrepancy identified as omission or addition, substitution, and change in dose, dosage form or route of administration, and frequency in each patient’s medication reconciliation process was considered as a key outcome variable. As a result, this medication reconciliation study considered medication discrepancy as any form of difference between the medication use history before transition and the transition or discharge medication orders. The reconciliation process was continued for all eligible patients until the required sample size was reached.

The process of medication reconciliation for each patient had two steps. In the first step, medication profile of each patient was registered into the data abstraction format. By the second step, medication profile of the patient was registered for orders at transition in care. As a result, medication profile for medication orders recorded at transition was reconciled by comparing with the formerly recorded medication profile for the same patient. Through such repeated medication reconciliation processes, all medications for the 411 patients were reconciled so that any type of discrepancy related to drug name, dose, dosage form, and frequency of administration was identified. All discrepancies identified through such reconciliation processes were repeatedly reviewed with the prescribing clinical teams in each unit and the teams were asked to justify whether the identified discrepancies were intentional or unintentional. Those discrepancies indicated by the prescribing teams with justifiable reasons were considered as intentional while discrepancies lacking such logical reasons were assumed unintentional. Consequently, unintended medication discrepancies were identified as medication errors.

#### Statistical analysis

The reconciled data were analyzed by use of Statistical Package for Social Sciences (SPSS), version 20.0 (IBM statistics, Armonk, NY, United States). Percent and frequency were employed to describe magnitude of medication discrepancy. Chi squared (χ^2^) test was conducted to identify potential relationship between medication discrepancy and explanatory variables. In addition, binary logistic regression analysis was carried out to identify factors associated with the medication discrepancy.

### Results

This study enrolled 411 patients to whom a total of 1027 medications were prescribed. More detailed sociodemographic characteristics of the enrolled patients can be referred from Additional file 1: Table S1. Characteristics of the enrolled patients showed that a slightly more medication discrepancy was occurred among females (53.8%) and it was more frequent among patients aged between 5 and 35 years (21.8–23.5%). Antimicrobial agents and cardiovascular drugs were found to have higher magnitude of medication discrepancy (140 and 106, respectively) compared to other categories (Table 1 and Additional file 2: Fig. S1).

**Table 1:** Characteristics of patients enrolled for medication reconciliation by discrepancy status at Hiwot Fana Specialized University Hospital, February–May, 2017

Characteristics	Categories	Medication discrepancy status	
		No discrepancy—frequency (%) (n = 292)	Had at least one discrepancy—frequency (%) (n = 119)
Sex	Male	127 (43.5)	55 (46.2)
	Female	165 (56.5)	64 (53.8)
Age (years)	< 5	54 (18.5)	19 (16.0)
	5–14	38 (13.0)	28 (23.5)
	15–25	63 (21.6)	26 (21.8)
	26–35	79 (27.1)	26 (21.8)
	36–65	23 (7.9)	8 (6.7)
	> 65	35 (12.0)	12 (10.5)
Religion	Muslim	246 (84.2)	98 (82.4)
	Orthodox	33 (11.3)	4 (3.4)
	Protestant	13 (4.5)	17 (14.3)
Residency	Urban	108 (37.0)	42 (35.3)
	Rural	184 (63.0)	77 (64.7)
Marital status	Single or under age	110 (37.7)	53 (44.5)
	Married	163 (55.8)	64 (53.8)
	Divorced	10 (3.4)	2 (1.7)
	Widowed	9 (3.10)	0 (0.0)
Educational level	Illiterate or basic	182 (62.3)	75 (63.0)
	Primary	83 (28.4)	34 (28.6)
	Secondary or higher	27 (9.2)	10 (8.4)
Ethnicity	Oromo	231 (79.1)	96 (80.7)
	Amhara	24 (8.2)	14 (11.8)
	Adare	23 (7.9)	3 (2.5)
	Others^a^	14 (4.8)	6 (5.1)
Ward of admission	Medical	81 (27.7)	54 (45.4)
	Pediatric	92 (31.5)	44 (37.0)
	Obstetrics and gynecology	45 (15.5)	9 (7.6)
	Surgical	74 (25.3)	12 (10.1)
Reason for admission	Respiratory and infectious diseases	83 (28.4)	32 (26.9)
	Cardiovascular diseases	15 (5.1)	34 (28.6)
	Malnutrition diseases	40 (13.7)	12 (10.1)
	Obstetrics and Gynecologic diseases	70 (24.0)	12 (10.1)
	Surgical and accidental diseases	31 (10.6)	5 (4.2)
	Others^b^	53 (18.2)	24 (20.2)
Medications	Antimicrobials and drugs for wound care	420 (75.0)	140 (25.0)
	Cardiovascular drugs	18 (15.0)	106 (85.0)
	Drugs for malnutrition therapy	91 (95.8)	4 (4.2)
	Hematologic drugs	68 (90.7)	7 (9.3)
	Drugs for liver disease therapies	33 (73.3)	12 (26.7)
	Obstetric and gynecologic therapies	32 (91.4)	3 (8.6)
	Others^c^	71 (73.2)	26 (26.8)
Duration of hospitalization (days)	< 5	150 (51.4)	18 (15.3)
	5–10	113 (38.7)	66 (55.9)
	11–15	25 (8.6)	26 (22.0)
	> 15	4 (1.4)	8 (6.8)

^a^Minor ethnic groups to the study area (e.g., Tigrie, Guraghe, Argoba, Somali, Sidama, etc.)

^b^Renal, liver, hematologic, gastrointestinal, and endocrine diseases

^c^Drugs used to treat respiratory, renal, gastrointestinal and endocrine disease conditions

During the process of medication reconciliation, the identified medication discrepancies totaled to 298 (29%) from the medications prescribed. Alongside, 293 (28.5%) of the discrepancies occurred at transitions in care while only 5 (0.5%) of the discrepancies were identified at discharge. The most frequent type of discrepancy identified was medication omission (13.9%); this was followed by discrepant doses (11.7%). However, majority of the medication discrepancies reconciled were intentional (19.6%) with only 9.4% of the medication discrepancies identified as unintentional (Table 2).

**Table 2:** Magnitude of medication discrepancy identified through medication reconciliation at Hiwot Fana Specialized University Hospital, February–May, 2017

Medication discrepancy	Frequency (n = 298)	Proportion (% within)
*Number of discrepant medications identified*		
At transition	293	28.5 (98.3)
At discharge	5	0.5 (1.7)
*Type of discrepancy identified*		
Omission	143	13.9 (48.0)
Discrepant dose	120	11.7 (40.3)
Discrepant dosage form	23	2.2 (7.7)
Discrepant frequency	12	1.2 (4.0)
*Reason for discrepancy*		
Intentional	202	19.6 (67.8)
Unintentional	96	9.4 (32.2)

The number in parenthesis indicates within percentage

Following univariate analysis, binary logistic regression analysis revealed that certain characteristics had a significant association with medication discrepancy. Accordingly, the magnitude of medication discrepancy was significantly reduced among patients admitted to surgical ward (adjusted odds ratio {AOR} [95% CI] 0.27 [0.10–0.74]) compared to patients admitted to medical ward. The magnitude of medication discrepancy was significantly increased to more than 5-fold (AOR [95% CI] 5.69 [2.4–13.62]) and decreased by 0.13-fold (AOR [95% CI] 0.13 [0.03–0.52]) which was specifically linked to patients who received drugs for cardiovascular diseases and malnutrition treatments, respectively, compared to those who received antimicrobial agents and drugs for wound care. Finally, the likelihood of medication discrepancy was significantly increased among patients who were hospitalized for more than 5 days (AOR [95% CI] 5.69 [2.97–10.9] {for 5–10 days of hospitalization}; 9.11 [3.84–21.63] {for 11–15 days of hospitalization}; and 28.0 [6.17–127.0] {for greater than 15 days of hospitalization}) compared to patients who were hospitalized for less than 5 days (Table 3).

**Table 3:** Factors associated with the medication discrepancy at Hiwot Fana Specialized University Hospital, February–May, 2017

Variable	COR [95% CI]	P-values	AOR [95% CI]	P-values
*Ward of patient admission*				
Medical	1		1	
Pediatric	0.76 [0.46–1.25]	0.28	1.73 [0.53–5.68]	0.36
Surgical	0.27 [0.12–0.59]	0.001	0.27 [0.10–0.74]	0.01*
Obstetrics and gynecology	0.24 [0.12–0.49]	< 0.001	0.59 [0.22–1.57]	0.29
*Drug category*				
Antimicrobials and drugs for wound care	1		1	
Cardiovascular drugs	6.64 [3.3–13.2]	< 0.001	5.69 [2.4–13.62]	< 0.001*
Drugs for malnutrition treatment	0.23 [0.07–0.79]	0.02	0.13 [0.03–0.52]	0.004*
Hematologic drugs	0.55 [0.20–1.49]	0.24	0.39 [0.11–1.41]	0.15
Drugs for liver disease treatment	1.74 [0.64–4.69]	0.27	1.07 [0.32–3.56]	0.91
Drugs for obstetric conditions	0.21 [0.03–1.64]	0.14	0.25 [0.03–2.33]	0.22
Others^a^	1.23 [0.53–2.85]	0.63	1.38 [0.49–3.87]	0.54
*Duration of patient hospitalization (days)*				
< 5	1		1	
5–10	4.86 [2.74–8.65]	< 0.001	5.69 [2.97–10.9]	< 0.001*
11–15	8.67 [4.16–18.0]	< 0.001	9.11 [3.84–21.63]	< 0.001*
> 15	16.6 [4.56–60.9]	< 0.001	28.0 [6.17–127.0]	< 0.001*

* Statistically significant difference

^a^Drugs used to treat respiratory, renal, gastrointestinal and endocrine conditions

### Discussion

Medication errors resulting from unintended medication discrepancies are among the primary cause of morbidity among hospital inpatients and need to be resolved through reconciliation [19]. This medication reconciliation study identified 298 medication discrepancies. Almost all medication discrepancies occurred at transition; one-third of them had no justification; greater proportion of them identified in medical and pediatric wards. Discrepancies were more likely to occur among patients prescribed with cardiovascular drugs and with prolonged time of hospitalization. All of these issues are the focus of this interpretation and are discussed separately.

In this pharmacist-led medication reconciliation processes at care transitions and discharge, 29% of medication discrepancies were identified and 32.2% of the discrepancies did not have justification. Similar studies also identified prescription medication discrepancy of 40.3% [25] and indicated a 25% reduction of potential errors related with medication discrepancy [5]. In addition, up to 30% reduction of reconciliation errors was also explained [26, 27]. Clinical pharmacist-led medication reconciliation indicated a 43.7% reduction of unintended medication discrepancy as well [28]. However, despite the potential of pharmacists in identifying and reducing medication discrepancy related errors, variable rates of benefits were highlighted in different settings [5, 25, 27–30], yet maintaining these accuracies remain as a challenge [29].

The most frequent type of medication discrepancy identified by this study was drug omission (13.9% from the total drugs reconciled). This magnitude was 49.3% from the total discrepancies identified. Consistently, a telephone-based medication reconciliation study found an omission rate of 38.6% from amongst the discrepancies [15]. A similar evidence in another study also showed an omission extent of 68% from within the discrepancies identified [7]. Moreover, despite variable percent of its extent, omission is still the most frequent type of discrepancy [31].

A higher magnitude of medication discrepancy was identified amongst medications received in medical and/or pediatric wards compared to medications received amongst surgical patients. Similarly, evidences of more frequent reconciliation error in medical wards even in teaching hospitals were also indicated [26, 31]. In agreement with other reconciliation studies, cardiovascular drugs were the most frequent class of drugs linked with the medication discrepancies identified through our reconciliation study [32–34].

The medication discrepancies were 5.69, 9.11, and 28 times more likely to occur among patients hospitalized for 5–10, 11–15 and > 15 days, respectively, compared to patients hospitalized for less than 5 days. Each intentional change by the prescriber during patient hospitalization should be well documented in medication plan aligned by a hypothesis that patients may increase their regimen changing behaviors when hospitalized for prolonged time; both of them could result into a high likelihood of medication discrepancy along with the increasing time of hospital stay [35].

Despite the presence of some study reports showing the association [2, 21, 23], binary logistic regression analysis did not show any potential relationship of age or sex with medication discrepancy in this study. Indeed, demographics of patients revealed comparable characteristics for sex and age (Table 1). However, repeated admissions in the past 12 months was not measured by our analysis due to record inconsistencies.

### Conclusions

The present study found that one-third of the medication discrepancies identified had no justification. The medication discrepancies were more likely to occur in medical and pediatric wards, with cardiovascular drugs, with patients hospitalized for more than 5 days. Therefore, a routine pharmacist-led medication reconciliation process benefits the medical and pediatric wards with a special focus on cardiovascular drugs. In addition, timely and plausible suggestion by healthcare professionals is important to reduce regimen changing behaviors of patients while also giving distinct consideration to patients hospitalized for more than 5 days.

## Limitations

Although significant number of medications were included for the medication reconciliation, this study had some limitations. First, some infectious disease management approach of the hospital is empiric or syndromic and this could overestimate the magnitude of intentional discrepancy. Second, since the study was conducted in selected wards where medications could be initiated at low doses and increased based on the patient response, such practices in these settings could also overestimate intentional discrepancy. Therefore, interpretation of the findings in this study should be made in consideration of these limitations.

## Additional files

**Additional file 1: Table S1.** Sociodemographic characteristics of patients enrolled for medication reconciliation at Hiwot Fana Specialized University Hospital, February–May, 2017. This table is a highlight for sociodemographic characteristics of the patients enrolled to the study for whom the medication reconciliation analysis was carried out. This characteristics description is about the patients enrolled and does not imply categorization between patients who had any type of medication discrepancy and those who had no discrepancy.

**Additional file 2: Fig. S1.** Magnitude of medication discrepancy identified by categories of medications reconciled at Hiwot Fana Specialized University Hospital, February–May, 2017. This figure highlights categories of medications reconciled with their respective count of identified discrepancy in each category. Antimicrobial agents or drugs used for wound healing and cardiovascular drugs were the frequent medications reconciled. The two medication categories showed the highest counts of discrepancy.

## Abbreviations

AOR: adjusted odds ratio
CI: confidence interval
COR: crude odds ratio
HFSUH: Hiwot Fana Specialized University Hospital
SPSS: Statistical Package for Social Sciences
