# Supplementary material for: Magnitude and factors associated with medication discrepancies identified through medication reconciliation at care transitions of a tertiary hospital in eastern Ethiopia
Source: BMC Res Notes. 2018 Aug 3;11:554. doi: 10.1186/s13104-018-3668-z (PMC6076390; doi:10.1186/s13104-018-3668-z)
Supplement: Supplementary file 1 — Additional file 1: Table S1. Sociodemographic characteristics of patients enrolled for medication reconciliation at Hiwot Fana Specialized University Hospital, February–May, 2017. This table is a highlight for sociodemographic characteristics of the patients enrolled to the study for whom the medication reconciliation analysis was carried out. This characteristics description is about the patients enrolled and does not imply categorization between patients who had any type of medication discrepancy and those who had no discrepancy. [file 13104_2018_3668_MOESM1_ESM.docx]

**Additional file 1: Table S1: Sociodemographic characteristics of patients enrolled for medication reconciliation at Hiwot Fana Specialized University Hospital, February - May, 2017.**

| Characteristics | Categories | Frequency (%) |
| --- | --- | --- |
| Gender | Male  Female | 182 (44.3)  229 (55.7) |
| Age (years) | <5  5-14  15-25  26-35  36- 65  >65 | 73 (17.8)  66 (16.1)  89 (21.7)  105 (25.5)  64 (15.5)  14 (3.4) |
| Religion | Muslim  Orthodox  Protestant | 344 (83.7)  50 (12.2)  17 (4.1) |
| Residency | Urban  Rural | 150 (36.5)  261 (63.5) |
| Marital status | Single or under age  Married  Divorced  Widowed | 163 (39.7)  227 (55.2)  12 (2.9)  9 (2.2) |
| Educational level | Illiterate or basic  Primary  Secondary or higher | 257 (62.5)  117 (28.5)  37 (9.0) |
| Ethnicity | Oromo  Amhara  Adare  Others* | 327 (79.8)  38 (9.2)  26 (6.3)  20 (4.9) |
| Ward of admission | Medical  Pediatric  Obstetrics and gynecology  Surgical | 135 (32.8)  131 (31.9)  86 (20.9)  59 (14.4) |
| Reason for admission | Respiratory and infectious diseases  Cardiovascular diseases  Malnutrition diseases  Obstetrics and Gynecologic diseases  Surgical and accidental diseases  Others** | 113 (27.5)  49 (11.9)  52 (12.7)  82 (20.0)  38 (9.2)  77 (18.7) |
| Duration of hospitalization | < 5 days  5-10 days  11-15 days  > 15 days | 168 (40.9)  179 (43.6)  51 (12.4)  12 (2.9) |

Others* stands for minor ethnic groups to the study area (e.g., Tigrie, Guraghe, Argoba, Somali, Sidama, etc.); others** stands for renal, liver, hematologic, gastrointestinal, and endocrine diseases.
