# Supplementary material for: Magnitude and factors associated with medication discrepancies identified through medication reconciliation at care transitions of a tertiary hospital in eastern Ethiopia
Source: BMC Res Notes. 2018 Aug 3;11:554. doi: 10.1186/s13104-018-3668-z (PMC6076390; doi:10.1186/s13104-018-3668-z)
Supplement: Supplementary file 2 — Additional file 2: Fig. S1. Magnitude of medication discrepancy identified by categories of medications reconciled at Hiwot Fana Specialized University Hospital, February–May, 2017. This figure highlights categories of medications reconciled with their respective count of identified discrepancy in each category. Antimicrobial agents or drugs used for wound healing and cardiovascular drugs were the frequent medications reconciled. The two medication categories showed the highest counts of discrepancy. [file 13104_2018_3668_MOESM2_ESM.docx]

## Additional file 2: Figure S1: Magnitude of medication discrepancy identified by categories of medications reconciled at Hiwot Fana Specialized University Hospital, Feb-May, 2017

*Others* stands for drugs used to treat respiratory, renal, gastrointestinal & endocrine disease conditions*
